# Novel Nano-Filled Coatings for the Protection of Built Heritage Stone Surfaces

**DOI:** 10.3390/nano11020301

**Published:** 2021-01-25

**Authors:** Mariateresa Lettieri, Maurizio Masieri, Mariaenrica Frigione

**Affiliations:** 1 CNR–SPIN, SuPerconducting and Other INnovative Materials and Devices Institute, via Giovanni Paolo II 132, 84084 Fisciano, Salerno, Italy; 2 CNR–ISPC, Istituto di Scienze del Patrimonio Culturale, Prov.le Lecce-Monteroni, 73100 Lecce, Italy; maurizio.masieri@cnr.it; 3 Department of Innovation Engineering, University of Salento, Prov.le Lecce-Monteroni, 73100 Lecce, Italy; mariaenrica.frigione@unisalento.it

**Keywords:** stone protection, nano-filled polymeric coatings, hydrophobic treatments, oleophobicity, anti-graffiti coatings, acrylic-based paints, guano simulation

## Abstract

An experimental nano-filled coating, based on a fluorine resin containing SiO_2_ nano-particles, was applied on calcareous stones, representative of materials used in buildings and monuments of the Mediterranean basin; for comparison purposes, two commercial products were applied on the same substrates. The efficacy of the protective treatments was assessed by analyzing different characteristics of the three experimental/commercial products, i.e., color changes and permeability to water vapor to evaluate the treatments’ harmlessness; capillary water absorption and water stone contact angle to evaluate the protection against water ingress; oleophobicity of the treated surfaces and the behavior under staining by acrylic blue-colored spray paint and felt-tip marker to verify the anti-graffiti action. Finally, the properties of the treated stone surfaces were analyzed also after the application of pancreatin, used to simulate bird excreta (guano). The protective coatings were found to promote graffiti removal, reducing also the detrimental effects due to simulated guano. The experimental nano-filled product, in addition, was able to provide outstanding performance but using smaller amounts of product in comparison to commercial systems.

## 1. Introduction

The protection of stone materials, belonging to ancient buildings and monuments and exposed to environmental agents and atmospheric pollution, is a permanent concern in the Cultural Heritage field. The application of nanotechnologies has allowed obtaining products with interesting and enhanced properties. New conservation strategies and innovative systems, in particular those based on inorganic nanoparticles added to organic matrices, aimed at minimizing the unwanted alterations (both environmental and anthropic) of the surfaces, are being constantly developed [1,2,3].

In the last few years, the application of materials providing multipurpose properties (such as hydrophobicity and anti-graffiti protection) has been strongly promoted to provide sustainable treatments, reduce maintenance costs and minimize restoration actions. Therefore, in addition to widely used hydrophobic coatings [4,5,6,7,8,9,10,11], surface treatments for stone have been tested in relation to their ability to reduce damages arising from several decay factors. On the other hand, physical, chemical, and biological processes may act, in isolation or in combination, yielding to the loss of surface integrity or even to structural failure. To provide protection against graffiti, many polymer-based coatings, displaying suitable properties (mainly oleo/hydrophobicity), have been formulated [12,13,14,15,16], but actually the anti-graffiti action needs to be proved on the particular stone material requiring protection. Biodeterioration caused by birds is another main cause of damage for stone materials in buildings, statues, and monuments [17,18], especially in historic city centers. Nevertheless, few studies in the literature have been devoted to the durability of protective coatings for stone under the action of bird excreta.

To address these issues, the University of Salento and CNR, both having expertise in the investigation of materials and treatments for the protection of surfaces in the built environment, have undertaken a wide research project on innovative products, such as nano-filled ones, able to protect stone surfaces from different agents.

In the present work, an experimental formulation, based on fluorine resins and SiO_2_ nanoparticles, was tested on Lecce stone and Trani stone, calcareous materials with different porosity. To evidence the advantages of this nano-filled system, two commercial protective products were tested for comparison: the first one is a similar product from the chemical point of view (i.e., a fluorine-based polymer) but without nano-particles; the second is a siloxane system, which is one of the classes of products most effective and frequently applied for stone protection, especially in the Cultural Heritage field. The treatments’ harmlessness was assessed by quantifying permeability and color changes. Contact angle measurements were used to evaluate the wettability and oleophobicity of the treated surfaces. Water absorption tests allowed quantifying the protection efficacy against water ingress. The behavior under staining by acrylic blue-colored spray paint and felt-tip marker were also investigated to verify the anti-graffiti action. Finally, the properties of the stone surfaces were assessed after the application of pancreatin, a mixture of digestive enzymes, used to simulate bird guano.

## 2. Materials and Methods

### 2.1. Materials

An experimental formulation and two commercial products, specially designed to provide hydrophobicity, dirt-repellence, and anti-graffiti properties to stone surfaces, were tested. The experimental product nanoF (manufactured and supplied by Kimia S.p.A., Perugia, Italy) is a water-based fluorine resin containing 10 wt% of SiO_2_ nanoparticles, 40–50 nm in dimensions (according to data provided by the supplier). A commercial fluorine-based product (trademark Fluoline PE, supplied by C.T.S. S.r.l., Altavilla Vicentina, Italy), hereinafter designated as F, was chosen to compare a chemically equivalent commercial formulation. For comparison purposes a second commercial product (trademark Kimistone DEFENDER, also supplied by Kimia S.p.A.), hereafter referred to as SW, was selected among silicon-based formulations, i.e., the most widely used protective materials for stone conservation. Data about the three protective systems are reported in Table 1. Further details about the applied products can be found elsewhere [19].

In a previous study [19], the viscosity of the used products was measured by rheological tests in steady state mode. A pseudo-plastic behavior was observed for all the investigated products and the viscosity values were similar to each other. The displayed rheological behavior confirmed that the products are suitable to be applied by brush and to provide an appropriate penetration into the stone materials.

Two stone materials with different porosity features were used as the substrates to test the three protective products, namely: “Lecce stone” (PS), a highly porous calcarenite with open porosity of 42% [20], and “Trani stone” (CS), a compact limestone with open porosity of 2% [20]. Both originating from quarries located in the Apulia region (south-eastern Italy), these stone materials are widely employed in civil and historical buildings. “Lecce stone”, typical of the Baroque architecture in the south-eastern Italy, exhibits properties and characteristics similar to those of porous materials used in many European countries (e.g., Malta [21], France [22], Portugal [23]). “Trani stone” was used in several buildings and monuments throughout Italy (such as Castel del Monte, a medieval site on the UNESCO World Heritage List) [24,25].

Stone samples of 5 × 5 × 1 cm^3^ dimensions were cut from quarry blocks using a saw; they were then smoothed with abrasive paper (180-grit), cleaned with a soft brush and washed with deionized water. The stone specimens were then dried in an oven at 60 °C, until a constant dry weight was achieved, and stored in a desiccator with silica gel (relative humidity, R.H. = 15%) at 23 ± 2 °C.

The materials used as staining agents were: blue-colored (RAL code 5015) acrylic spray paint in pressurized can (Cilvani RAL made by Cilvani S.r.l., Caivano, Italy); blue-colored (RAL code 5005) water-based acrylic paint marker (POSCA by UNI Mitsubishi pencil, Tokyo, Japan), with a 1.8–2.5 mm wide bullet tip (PC-5M); pancreatin (Carlo Erba Reagents S.r.l., Cornaredo, Milan, Italy), provided as a powder and dissolved in an aqueous solution.

### 2.2. Treatments

**Figure 1:**
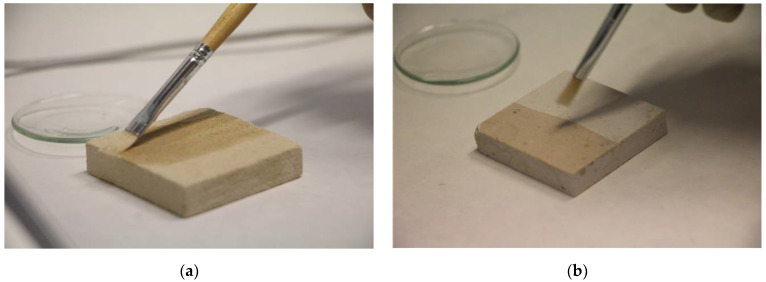
Application by brush of the nanoF product on samples of (**a**) porous stone (PS samples) and (**b**) compact stone (CS samples).

The protective coatings were applied by brush on the 5 × 5 cm^2^ side of each specimen; examples are illustrated in Figure 1.

According to preliminary tests [19], different amounts of product were used: 150 g/m^2^ of nanoF, 150 g/m^2^ of F, or 300 g/m^2^ of SW for PS; 50 g/m^2^ of nanoF, 50 g/m^2^ of F, or 100 g/m^2^ of SW for CS. After the application, the specimens were kept in the laboratory (at 23 ± 2 °C and 45 ± 5% R.H.) for 30 days and dried in oven at 40 °C for 7 days.

Staining with acrylic blue-colored paint, either as spray or felt-tip marker, was carried out on untreated and protected stone samples. Two coats of paint were sprayed on specimens placed on a 45° tilted surface. To limit paint deposition to an area of 1.5 × 5 cm^2^, the staining was performed with a stencil and the lateral sides of the specimens were protected with a PET film. After the application of the paint, the samples were stored for 2 days in laboratory conditions (23 ± 2 °C, 50 ± 5% R.H.). On the same samples, staining with the marker was also performed, covering an area of approximately 1.5 × 5 cm^2^. The removal of both staining agents was carried out 20 days after the staining, using analytical grade acetone (Carlo Erba Reagents, Cornaredo, Milan, Italy). As recommended by the current standard [26], a wet paper towel was rubbed across the stained area for 25 complete back and forth wipes, dunking the towel in acetone every five cycles.

To reproduce the effects of bird-dropping (guano), the stone samples were stained using an aqueous solution of pancreatin (1:20) [27]. An area 5 × 5 cm^2^ of each untreated and protected specimen was smeared with the pancreatin solution. The cleaning by running tap water and a sponge was performed 30 days after the staining. Finally, the samples were dried in laboratory conditions for 20 days and, then, in oven at 40 °C for 3 days. In the text below, the term “pancreatin test” will be referred to the complete procedure (i.e., pancreatin application, cleaning, and drying).

Each treatment was performed on sets of three PS and three CS specimens; separate sets (three specimens for each stone) without treatment were tested as a control reference. Before the application of each product, the stone specimens were conditioned 24 h in laboratory conditions (23 ± 2 °C, 45 ± 5% R.H.), to achieve an equilibrium with the surrounding environment.

### 2.3. Measurements and Tests

Color measurements [28] were carried out with a CM-700d spectrophotometer (Konica Minolta Sensing, Singapore) using a CIE Standard illuminant D65 and a target mask 8 mm in diameter. Ten measurements were performed on each specimen/area and the instrument was recalibrated to a white calibration cap at the start of each measurement session. The color coordinates expressed in the CIE L*a*b* color space (1976) were collected and the color difference (ΔE*_ab_) was calculated using the following equation:

ΔE*_ab_ = [(ΔL*)^2^ + (Δa*)^2^ + (Δb*)^2^]^1/2^, (1)

The color variations were calculated in comparison to the untreated surfaces; only for the surfaces subjected to the pancreatin test, the changes in color were evaluated in comparison to the surfaces before the staining.

The changes in water vapor permeability were investigated by vapor transmission test [29] performed at 20 °C in a climatic chamber (ACS Angelantoni Climatic Systems, Mod. UY 600, Massa Martana, Perugia, Italy). Weight measurements were carried out every 24 h and the mass changes (ΔM) were calculated as the average of five consequent values of the daily difference in weight. Then, the reduction in water vapor permeability was quantified as follows [29]:

ΔVP = [(ΔM_bt_ − ΔM_at_)/ΔM_bt_] × 100, (2)

where ΔM_bt_ and ΔM_at_ are the weight changes in the steady state for the samples before and after the treatment, respectively.

Static contact angles were measured at different positions of the sample surface using either water [30] or oil [19] as wetting liquids. A Costech apparatus (Costech International S.p.A., Cernusco sul Naviglio, Milan, Italy) was used to deposit microdrops and to record the shape of the drops (15 s after its deposition) with a camera. For each specimen, the contact angles are the averaged results of 30 and 5 measurements for water and oil liquids, respectively.

The capillary water absorption was evaluated following the procedure described in the European standard [31]. The test was carried out for 6 days. The amount of absorbed water (Qi) at each time of exposure was determined as follows:

Q_i_ = (w_i_ − w_0_)/A, (3)

where: w_i_ and w_0_ are the weight of the sample at time t_i_ and t_0_, respectively; A is the area exposed to water. Then, the protective efficiency (PE) was calculated from the amounts of water absorbed, using Equation (4):

PE = [(Q_0_ − Q_t_)/Q_0_] × 100, (4)

where: Q_0_ is the amount of water absorbed from the uncoated samples; Q_t_ the amount of water absorbed after the protective treatment or the pancreatin test.

The cleaning efficacy (CE), referred to the removal of spray paint or marker, was evaluated as a percentage by Equation (5):

CE = {1 − [(ΔE*_ab_)_cleaned_/(ΔE*_ab_)_stained_]} × 100, (5)

where: (ΔE*_ab_)_cleaned_ is the color variation of the cleaned surfaces; (ΔE*_ab_)_stained_ is the color variation of the stained surfaces and the ratio (ΔE*_ab_)_cleaned_/(ΔE*_ab_)_stained_ is the residual stain [32].

## 3. Results

### 3.1. Harmlessness of the Treatments

**Figure 2:**
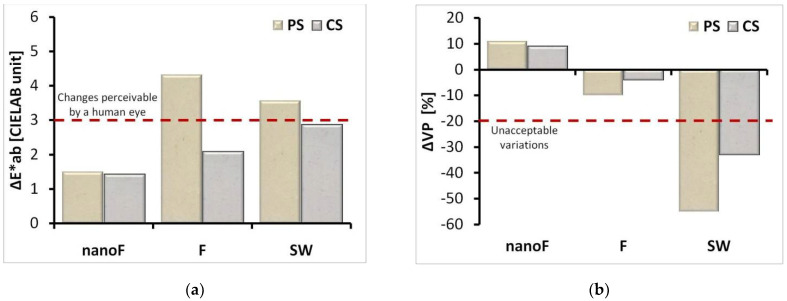
(**a**) Color changes (ΔE*_ab_) and (**b**) variations in water vapor permeability before and after the products’ application. The columns’ colors reproduce the appearance of the stone.

The application of the nanoF product did not produce significant color changes on either the porous or compact stone surfaces (Figure 2a) with ΔE*_ab_ of approximately 1.5 CIELAB units. On the other hand, in the samples treated with both F and SW products large color variations were observed, being ΔE*_ab_ close or higher than 3, but lower than 5.

As illustrated in Figure 2b, very high reductions in water vapor permeability were measured in the samples treated by the siloxane-based product (i.e., SW), with values greater than the acceptable threshold of 20% [33]. Small decreases in the same property were measured with the fluoropolyether-based formulation (i.e., product F), while unexpected increases in permeability were found after the application of the nanoF product. Such an interesting result was already observed in membranes [34] functionalized with highly hydrophobic thin coatings; stone materials with superhydrophobic [35] or nanocrystal-based polymer coatings [36,37] can exhibit a similar behavior. The increase in permeability was ascribed to the enhancement of water vapor diffusion through hydrophobic pores since the low surface energy of the coating allows preventing the water molecules to condensate on the pore walls [38]. This effect cannot be observed if the applied coatings, although highly hydrophobic, reduce the pore dimensions.

### 3.2. Oleo/Hydrophobicity of the Treated Stone Surfaces

**Figure 3:**
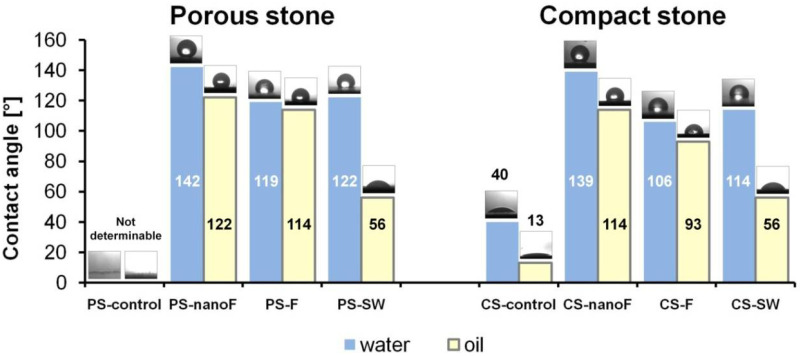
Contact angle values; the water and oil droplets on the stone surfaces are also shown.

The applied coatings lead to a significant reduction in surface wettability with respect to both water and oil, as resulting from the large growth of contact angle values measured on the stone surfaces after each treatment, observable in Figure 3. The greatest values of contact angle were obtained using the coatings with nanoparticles (i.e., nanoF), regardless of the stone type. The changes are particularly outstanding for the PS substrate, where, without any coating, the drops of liquid (water or oil) were suddenly soaked into the stone.

Lower contact angles were measured by dropping oil relative to water, due to the low surface tension of oil drops (32 mN/m [39]) in comparison to that of pure water ones (72 mN/m [40]). Taking into account that water-stone contact angles greater than 90° are typical of hydrophobic surfaces [41], while oil-stone contact angles above 70–80° are measured on oleophobic surfaces [42], almost all of the treated stone surfaces achieved good hydrophobicity and oleophobicity; only the SW product was not able to provide a high oleophobicity, irrespective to the stone substrate.

### 3.3. Protection Efficacy against Capillary Water Absorption

**Figure 4:**
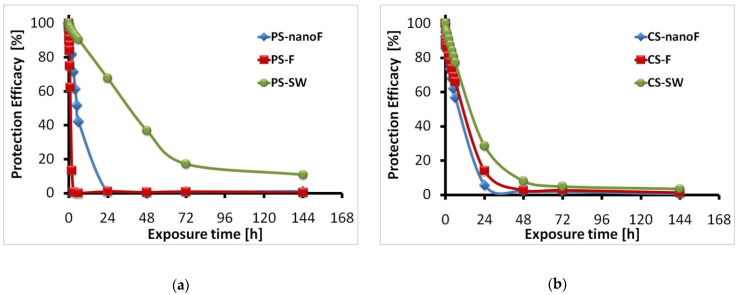
Protection efficacy (PE) against capillary water absorption as a function of exposure time measured on: (**a**) porous stone; (**b**) compact stone.

In all the treated samples, the protection against capillary water absorption was generally effective for few hours, while the protective action was almost completely lost within 24 h. Only PS treated with the SW system retained efficacy at longer times, probably due to the greater amount of SW product applied on the PS surfaces (Figure 4a). The protection efficacy rate for the CS material was almost the same irrespective of the applied coating (Figure 4b).

It is to highlight that the protection against capillary water absorption assured by the experimental nanoF product is comparable to that obtained using commercial protective systems without nanoparticles.

### 3.4. Assessment of the Anti-Graffiti Action

From the results illustrated in Section 3.2, it is possible to conclude that both the nano-filled product nanoF and the commercial F formulation are suitable candidates for graffiti protection. Treatments able to supply both hydrophobicity and oleophobicity to the stone surfaces are, in fact, expected to act as effective anti-graffiti systems since they minimize the contact between the substrate and applied inks or sprayed paints [43].

**Figure 5:**
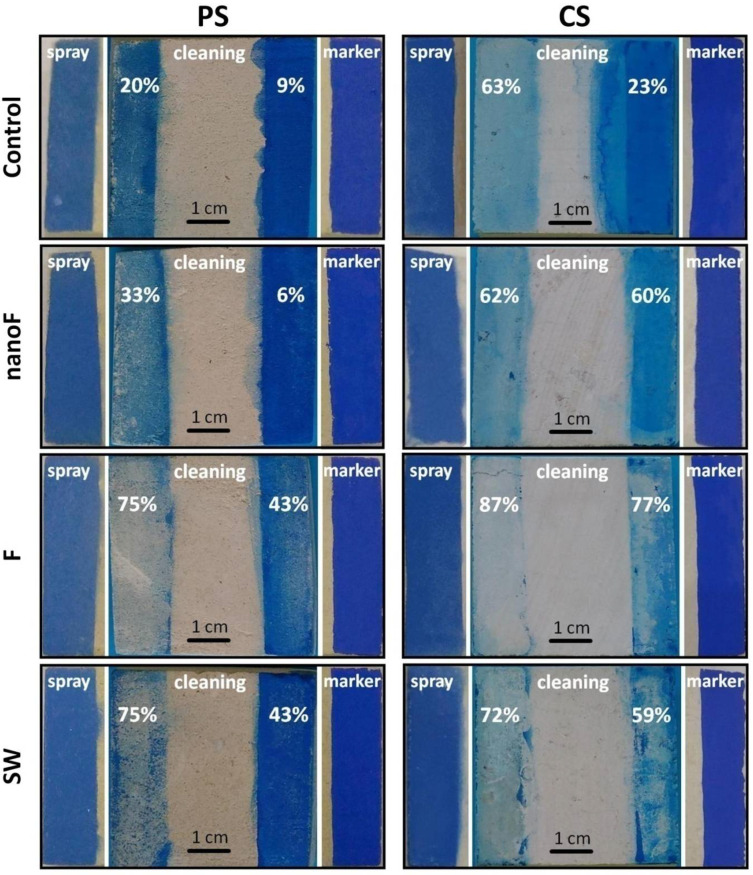
Stone samples after the staining and after the cleaning procedure; the CE values, as defined in Equation (5), are reported for the cleaned areas. The un-stained stone surface is in the middle of each image.

The evaluation to the visual inspection by the naked eye (Figure 5) already proved that the removal of the stained agents with acetone did not give successful results.

In the literature, several methods are reported to quantify the efficacy of stain removal based on the residual stain [32] or examining the ΔE*_ab_ after the cleaning [44]. Similarly, cleaning efficacy (CE) above 90% means effective stain removal while CE below 80% cannot be accepted; CE between 90% and 80% are tolerable values, although not optimal. Therefore, in the investigated samples, the cleaning with acetone resulted acceptable only for the CS-F specimens stained with spray paint, being CE = 87% for such couple. The removal of the stain applied by the felt-tip marker was even less efficient. However, markers are reported as the most aggressive staining agents among the methods for graffiti writings [45] due to their high solvents content which easily spreads the ink within the pores of the substrate. Nevertheless, the application of a protective treatment was helpful in facilitating the removal of the staining agent. As reported in Figure 5, the CE percentages were higher, or at least comparable, than those calculated for the unprotected specimens.

### 3.5. Durability to Simulated Bird Guano

The staining with the simulated guano and subsequent cleaning caused color changes to the investigated stone surfaces (Table 2). The protective coatings appeared to be less affected after the test, while the appearance of the unprotected control surfaces was changed to a greater extent. The surfaces treated with nanoF showed the lowest ΔE*_ab_.

After the pancreatin test, changes in permeability to water vapor (ΔVP) were also measured (Table 2). Good results were obtained for nanoF treated specimens, especially on the porous stone, even if variations exceeding the acceptable threshold of −20% were found in all the samples. In the SW-treated samples, a limited increase in permeability suggests that the barrier action of the coating was weakened after the pancreatin test. High decreases observed also for the unprotected stone materials suggested that pancreatin residues into the pores acted as a barrier to the movements of the water molecules inside the stone.

The wettability of the treated surfaces was little affected by the pancreatin test (Table 2). The nanoF-samples exhibited greater decreases in water-stone contact angles. Nevertheless, these variations are not significant since values still above 110° were measured for all the treated samples. Contrary to what was observed for the untreated porous stone, the contact angles were measurable also on the PS-control samples after the pancreatin test, once again suggesting the presence of pancreatin residues into the pores.

**Figure 6:**
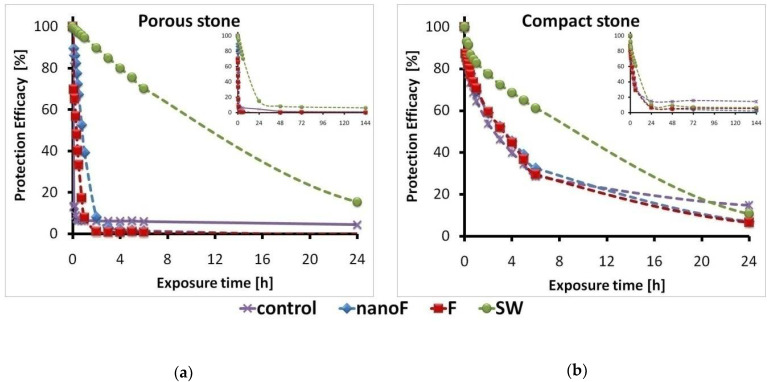
Protection efficacy (PE) against capillary water absorption as a function of exposure time measured after the pancreatin test on: (**a**) porous stone; (**b**) compact stone. The protection efficacy up to 6 days of exposure is reported in the inset boxes.

Although reductions in protective efficacy were found after the pancreatin test (Figure 6), the efficacy of the applied coatings against the ingress of liquid water by capillary action was preserved. Variations can be due to modifications of the coating; however, examining the PE values at the very short times of exposure, the barrier effect was still satisfactory. The protective efficacy observed for the control samples (both PS and CS) was a false effect that can be attributed to the incomplete removal of pancreatin.

## 4. Discussion

The treatments with the nanoF product yielded good results in terms of compatibility with the used stone materials. Color variations (i.e., ΔE*_ab_) lower than 3 were measured, that is the value considered perceptible by the human eye [46,47]. In fact, none of the treatments exceeded the threshold (ΔE*_ab_ ≤ 5) judged as tolerable in conservation interventions on monuments and built heritage [44,48]. The permeability changes are also very low. Actually, the application of the product with nanoparticles produced a small increase in permeability due to a higher hydrophobicity of the pore walls that enhance the water vapor diffusion through the stone. Unaffected permeability of the stone materials after the application of a protective coating on the surface is a beneficial result since decreases in this parameter may activate the material’s decay. If regions (or layers) with different water vapor permeability are present in the stone, water may condensate inside the pores generating detrimental mechanical stress; in particular, this phenomenon may occur at the interface between the untreated and treated parts with possible consequent detachments.

All the applied treatments decreased the wettability of the surface with respect to both water and oil. The highest contact angles were found on the surfaces treated with the nanomaterial (nanoF) irrespective of the porosity of the stone; values close to 120°and 140° were measured with oil and water, respectively. In fact, as reported in the literature [1], the addition of SiO_2_ nano-particles in polymer coatings can strongly reduce the wettability of the treated surfaces; in these cases, the contact angle values decrease since the nanoparticles are able to produce bioinspired nanostructures which minimize the area of contact between the liquid drops and the surface.

All the coated stone materials showed similar behaviors in the protective action against water ingress by capillarity. The presence of a coating reduced the water absorption in the early steps of the test, but the protective action was lost at longer times of exposure. The nanoparticles do not seem to give further beneficial effects, while the protective action increased where greater amounts of product were applied to obtain the superficial coating. The protective efficacy values seem to be in disagreement with the very high hydrophobicity previously observed for the coated specimens. On the other hand, a high protection efficacy is expected where low surface wettability (i.e., high contact angle values) is measured. However, the observed behavior is not uncommon [49,50]. Contact angle measurements and capillary water absorption are complementary tests and do not provide the same information: the capillary absorption test allows calculate the long-term water uptake through the entire area of the specimen; the static contact angle evaluates the punctual hydrophobicity (i.e., at the interface between the water drop and stone surface) at a very short time.

The presence of protective coatings did not prevent the stone surface from staining with either spray paint or felt-tip marker. Uniform colored films were observed on the surface and the applied staining agent totally hid the stone beneath. Where the protective coatings were applied, the cleaning with acetone allowed removing the paint to a certain extent but this procedure did not give acceptable results. The cleaning was more effective in the compact stone; on the other hand, the paint on a highly porous stone can be barely removed because it easily penetrates beneath the surface and is retained into the pores [32]. In addition, the results confirmed the stronger action of the felt-tip marker in comparison to other staining agents. It is worth noting that both hydrophobicity and oleophobicity of the treated materials were proved, then anti-graffiti action should be assured. In fact, the anti-graffiti performance was not satisfactory even in the surfaces treated with the coatings containing nanoparticles, where very high oleo/hydrophobicity was observed. Therefore, it can be argued that the actual effectiveness cannot be inferred a priori from the coating’s properties but experimental tests in the specific applicative conditions need to be performed.

The presence of coatings on the stone surfaces gave protection also against the action of bird dropping. The unprotected stone was affected to a greater extent than the coated materials, especially in terms of color and permeability. The treated stone retained its properties after exposure to the pancreatin. Superior initial performances of the nano-filled system allowed counteracting in a better way the damaging effects of the simulated guano.

To sum up, the application of the system containing the nanosized SiO_2_ yielded better performance in comparison to other common protective systems widely applied for the protection of monuments and built heritage. Amounts of the novel protective nanomaterials comparable or even lower than those of commercial products without nanoparticles can provide a good protective action. Such a result allows fulfilling the minimum intervention criteria required in the conservation of Cultural Heritage artifacts.

## 5. Conclusions

In this study, an experimental formulation, based on fluorine resins and SiO_2_ nano-particles, was tested on two calcareous stone materials (Lecce stone and Trani stone), having different porosity; the performance of the nano-filled coating was compared to those of two commercial protective products.

The nano-filled experimental product was able to act as an efficient protective surface treatment for compact/porous stone surfaces against water/oil ingress and also for graffiti staining. Low wettability and improved permeability were, in fact, observed; this latter result probably originated from the high hydrophobicity of the pore walls inside the stone. Despite the surface oleophobicity, the anti-graffiti efficacy was found to depend on the staining agent and cleaning procedure, rather than on the stained substrate; further trials to verify the efficacy of different cleaning methods are still in progress. The experimental coating was proved to be effective in providing suitable protection also against the degradative action of pancreatin. Ongoing tests devoted to durability under either artificial (in solarbox) or natural (outdoor) exposure will be the subject of a next study.

In conclusion, satisfactory properties were achieved using the experimental nano-filled product, which meets the requirements of stone conservation/protection for civil and historical buildings. Performance comparable or even greater than those displayed by commercial systems were obtained using smaller amounts of the nano-filled formulation. As a consequence, sustainability criteria, in particular in terms of costs and environmental impact, are fulfilled with the use of this novel nano-filled product.

## Figures and Tables

**Table 1:** Details of the used protective products (* data supplied by Kimia S.p.A.; ^§^ from the technical sheet).

Product	nanoF	F	SW
Polymer component	Fluorine resin (12.7 wt%) *	Fluoropolyethers (10 wt%) ^§^	Organic silicon compounds and microcrystalline waxes ^§^
Nanoparticles	SiO_2_ (10 wt%) Particles, 40–50 nm in dimensions *	---	---
Mixture	Water dispersion *	Water dispersion ^§^	Water solution ^§^
Appearance	Colorless transparent liquid	Slightly white transparent liquid	Milky white opaque liquid
Density [g/cm^3^]	1.04 *	1.05 ^§^	0.99 ^§^
pH	7–8 *	7 ^§^	7 ^§^

**Table 2:** Effects of the pancreatin test: color difference (ΔE*_ab_), changes in vapor permeability (ΔVP), and variations of contact angle values (Δα) in comparison to the surfaces before the staining.

Substrate	Protective Treatment	ΔE*_ab_ [CIELAB Unit]	ΔVP [%]	Δα [°]
Porous stone (PS)	Control	3.97	−34	+19
	nanoF	1.39	−30	−23
	F	3.30	−34	+3
	SW	1.81	+39	−2
Compact stone (CS)	Control	4.29	−68	−3
	nanoF	0.54	−36	−30
	F	0.59	−34	+6
	SW	0.64	+13	−1

## Data Availability

The data presented in this study are available on request from the corresponding author. The data are not publicly available as they form part of an ongoing study.
